# A Novel Ultrasonic Fatigue Test and Application in Bending Fatigue of TC4 Titanium Alloy

**DOI:** 10.3390/ma16010005

**Published:** 2022-12-20

**Authors:** Sen Tang, Xinyu Wang, Beihai Huang, Dongtong Yang, Lang Li, Chao He, Bo Xu, Yongjie Liu, Chong Wang, Qingyuan Wang

**Affiliations:** 1 School of Mechanical Engineering, Chengdu University, Chengdu 610106, China; 2 Failure Mechanics and Engineering Disaster Prevention Key Laboratory of Sichuan Province, Sichuan University, Chengdu 610065, China; 3 Institute for Advanced Study, Chengdu University, Chengdu 610106, China

**Keywords:** very high cycle fatigue, bending fatigue, fatigue test, titanium alloy

## Abstract

The very high cycle fatigue (VHCF) problems of thin-plate structures are usually caused by high-frequency vibrations. This paper proposes an accelerated fatigue test method based on ultrasonic loading technology in order to develop a feasible bending testing method and explore the bending fatigue characteristics of thin-plate structures in the VHCF regime. A new bending fatigue specimen with an intrinsic frequency of 20 kHz was designed based on cantilever bending through finite element simulation. It was verified by the axial load test with R = −1. The results showed that the method could effectively transfer the dangerous cross-section at the first-order cantilever bending restraint to the internal part of the specimen, thereby making the fracture location independent of the complex stresses. The linear relationship between the vibration amplitude and the maximum stress was also verified using strain measurements. Furthermore, the S-N curves and fracture morphology for different loading types were consistent with conventional studies to a certain extent, which indicated that the design of the bending test model was reasonable.

## 1. Introduction

As fatigue research has been progressively deepened, it has been recognized that materials and structures can be damaged by fatigue within 10^7^ to 10^9^ cycles, which involves the problem of VHCF [1,2]. In fact, aviation components such as engine blades are vulnerable to the combined action of high frequency and low load, which means their failure forms also have VHCF characteristics [3]. At the same time, the revised national military standard “General Specification for Aeronautical Turbojet and Turbofan Engines” (GJB241A-2010) also clearly stipulates that “all components of the aero-engine must have a life of not less than 10^9^ cycles”. Therefore, due to the higher requirements for long life and reliability of aviation components, the research on VHCF has received special attention in the aerospace field.

Benefiting from the development of piezoelectric transducer, the ultrasonic fatigue testing machine with a loading frequency of 20 kHz has achieved great success in axial loading experiments and has been initially explored in bending experiments [4,5,6,7,8]. It has gradually become the most popular method, and many typical fatigue mechanisms have been obtained in the VHCF regime [9,10,11,12]. However, it is often reported that differences in loading methods will lead to different fatigue results [13,14,15]. Although the fatigue data of axial loading can reflect the fatigue performance of materials and structures to a certain extent, the method of predicting bending life based on axial fatigue data may not be applicable due to the heterogeneity of materials and the difference in the stress of structures [16,17,18]. When comparing the typical high-frequency axial loading and rotary bending loading tests, it can be seen that rotary bending loading will make the fatigue data more discrete, which has been proved to be related to the heterogeneity of the materials (microstructure, holes, and inclusions) and non-uniform loading [19,20]. In addition, the rotary bending load can usually obtain a better fatigue performance at higher stress amplitudes, but with the decrease in stress amplitude, the difference in the fatigue life gradually decreases [21,22]. The S-N curves from bending and axial fatigue cross, which means the bending fatigue life will be overestimated if directly extended from high cycle fatigue (HCF) to VHCF. The crack is initiated from inner facts, and even a stress gradient exists in bending fatigue.

When extending to the VHCF regime, researchers have made many attempts to investigate the effect of loading methods on fatigue performance, mainly in three-point bending and cantilever bending. For example, Bao et al. [23] investigated the fatigue behavior of TC4 titanium alloy in HCF and VHCF regimes by using ultrasonic three-point bending. Macek et al. [24] studied the roughness distribution of 2017A-T4 aluminum alloy after bending fatigue by using an optical zoom surface-measurement technique. Wang et al. [25] investigated the high cycle and very high cycle fatigue properties of CFRP-aramid honeycomb sandwich structures in three-point bending using ultrasonic three-point bending. Hu et al. [26] investigated the bending fatigue behavior of 316 stainless steel using ultrasonic cantilever bending. Due to the immature testing scheme, the ideal results have not been obtained because there are many obvious disadvantages of the existing test scheme. Firstly, it takes a long time to get the experimental data due to the low loading frequency [19,20]. Secondly, due to the influence of complex stresses in the constrained position, the existing methods for studying very high cyclic bending fatigue cannot effectively solve the problem of cantilever bending fracture location [27]. The lack of corresponding very high cycle bending fatigue data causes great problems for the subsequent use and design of the corresponding materials and structures. Therefore, it is extremely important to develop an effective method for bending fatigue experiments based on ultrasonic loading.

To solve the above problems, this study proposed a method for bending tests of materials based on an ultrasonic fatigue test system. Aiming at TC4 titanium alloy commonly used in aero-engines, a new type of bending specimen was designed to effectively transfer the dangerous section of the restraint in cantilever bending to the gauge section of the specimen, and HCF and VHCF tests were respectively conducted. In addition, while considering the effect of loading type on the fatigue life test results, a set of ultrasonic axial fatigue tests was added. Finally, the two results were compared**.**

## 2. Specimen Design for Very High Cycle Bending Fatigue

The design of a bending fatigue specimen needs to consider factors that include the intrinsic frequency of the specimen, the vibration mode, and the stress distribution of the specimen [4,28]. In order to ensure that the ultrasonic fatigue test system can resonate at 20 kHz, it is desirable to ensure that the intrinsic frequency of each component of the test system (including amplifiers, extension bars, and bending specimens) is maintained near 20 kHz. The design of the specimen needs to consider the following. Firstly, the stress distribution within the specimen must be suitable for bending fatigue testing. Another consideration is the need to design an effective connection method to facilitate the installation of the specimen into the equipment. Finally, in order to control the stress amplitude ($\sigma_{a}$) via excitation displacement (*A*_0_), the coefficient of displacement to stress ($C_{s}$) needs to be calculated before the experiment [29]. Therefore, the bending vibration mode of the specimen as well as the distribution of stresses in the gauge section need to be considered.

### 2.1. Specimen Design and Analysis

Since there is no unified standard for the design of bending specimens, finite element analysis (FEA) is used as a common way for the design of non-standard parts. It is known from previous studies that the heat generated by friction can be effectively reduced by appropriately reducing the restraint area at the connection position [29], so the combination of double round holes and round spacers was still used for the restraint method of the specimen designed in this paper. A series of finite element models with different geometric shapes were designed by using the commercial finite element software ABAQUS (v6.14, Dassault systemes, Providence, RI, USA). In order to mount the specimen to the excitation head, two round holes with a 3.2 mm diameter were designed in the middle of the specimen (excitation area) where the localized stress potentially would become a fracture-risk area due to the stress concentration. Therefore, the gauge section was designed in-between a hyperbola section to guarantee the stress was 1.2 times greater than in the excitation area.

**Figure 1:**
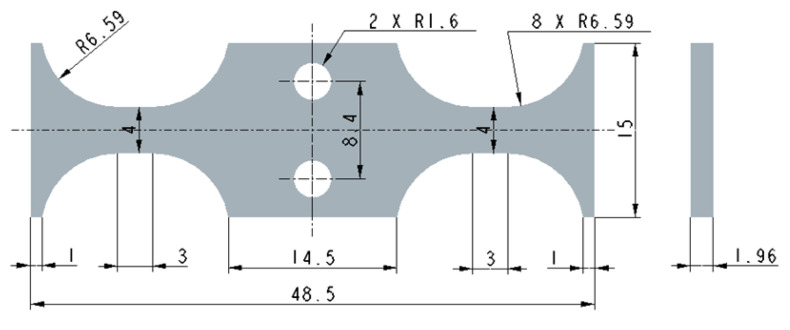
The geometry of the ultrasonic bending loading specimen (unit: mm).

After the finite element analysis and calculation, the intrinsic frequency of the specimen was found to be 19.99 kHz, which satisfied the resonance condition of the ultrasonic fatigue system. The geometry of the bending specimen was finally determined as shown in Figure 1. Two round holes in the middle of the sample were used to install screws so that the sample could be effectively connected to the end of the excitation rod. In addition, designing the gauge section as a rectangular section with a length-width ratio of 2:1 could effectively reduce the value of the maximum stress per unit amplitude and ensure the accuracy of the test data. Finally, the specimen was considered as a symmetric structure in order to ensure that an excellent distribution of stress existed in the specimen and to ensure that the dangerous cross-section was located in the specimen gauge section.

**Figure 2:**
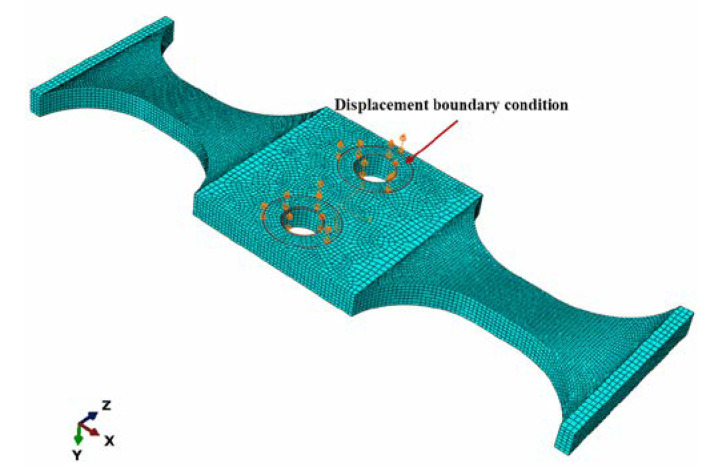
The finite element model of the specimen.

The final 3D finite element model is shown in Figure 2. Since the stress amplitude was much smaller than the yield strength of the material in the HCF and VHCF regime, the specimen was designed as a linear elastic solid; the basic properties of the material are referred to in the Materials section (as introduced in Section 3.1). As shown in Figure 2, a displacement constraint perpendicular to the specimen surfaces with amplitude of 1μm was set as a boundary condition of excitation displacement to obtain the $C_{s}$. The element type was set to C3D8R, which is a continuous, solid, 8-node, simplified integral element. The finite element analysis results were sensitive to the mesh density [30,31]. The denser meshes led to an accurate result but had a higher computational cost. Therefore, a suitable mesh density was needed to capture the mechanical effects accurately and at the same time to save costs. The global seed was set uniformly to 0.3. The total number of elements in the model was 48,475. The lanczos solver in ABAQUS software was used to find the intrinsic frequency of the specimen and also to obtain the stress distribution of the specimen.

**Figure 3:**
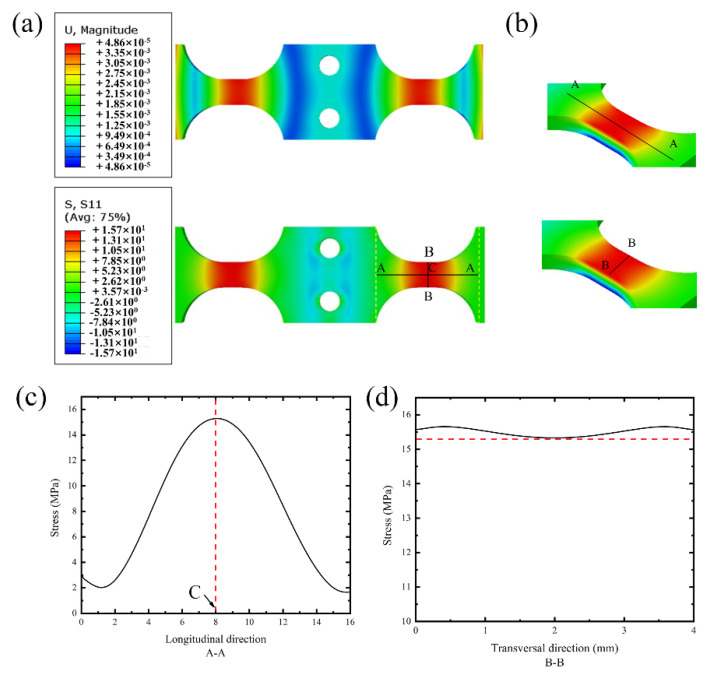
The stress distribution on the bent specimen: (**a**) finite element analysis (FEA) solved dis-tribution of displacement and stress; (**b**) paths of stress collecting at the gauge section; (**c**) stress profile along the longitudinal direction; (**d**) stress profile along the transversal direction.

In order to study the stress coefficient and stress distribution along the half-length of the specimen, the displacement and stress distribution clouds were obtained via the ABAQUS simulation analysis under a 1 μm displacement loading as shown in Figure 3a. Benefiting from the convenience of FEA, one of the gauge sections of the stress cloud was taken and its stress distribution curves were obtained along two specific orthogonal paths (A-A and B-B, respectively). As shown in Figure 3c, the point of maximum stress was located just in the middle of the A-A curve. In addition, the stress distribution along the B-B path had the highest stresses near the edges, but the overall variation was small, so the stress difference along the waist was negligible. The coefficient of displacement to the stress $C_{s}$ = 15.70 MPa was obtained via computational simulation in this specimen in a boundary condition of 1 μm excitation displacement. The secondary strain-X and displacement-Y distribution curves along A-A are shown in Figure S3 of the Supplementary Material.

Based on the finite element analysis, the novel design had the following advantages. Firstly, compared with the results of previous studies [29], the model of this symmetric structure effectively reduced the coefficient ($C_{s}$) from 125 MPa to 15.70 MPa, which effectively expanded the control interval of the ultrasonic fatigue system and further ensured the accuracy of the fatigue data. Secondly, compared to cantilever bending specimens [27,32], the specimen model effectively shifted the dangerous section from the constraint to the interior of the specimen, thereby simplifying the forces at the fracture location. Finally, compared to the three-point bending specimen [23,33,34], the model allowed for symmetric bending experiments with R = −1.

### 2.2. Stress Calibration

**Figure 4:**
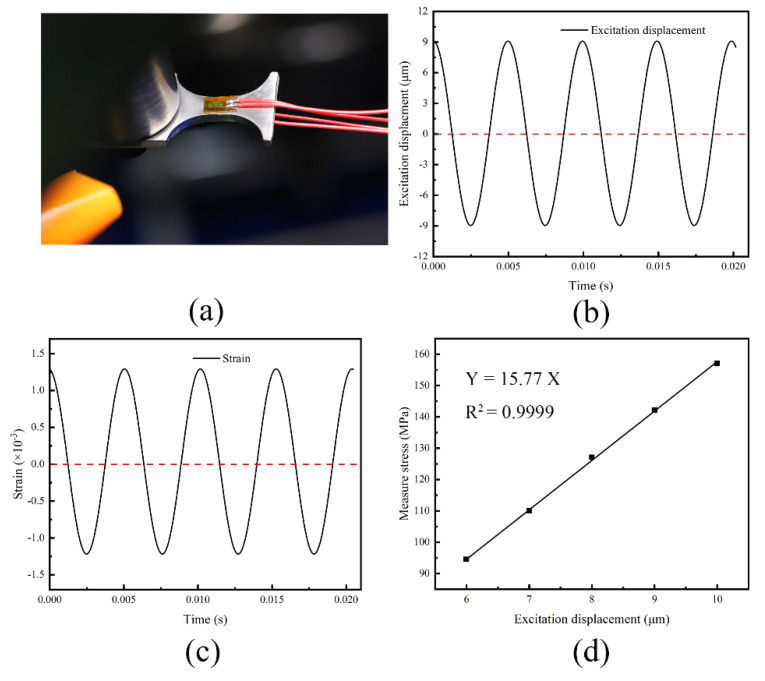
Strain measurement: (**a**) diagram of strain gages; (**b**) waveform of excitation displacement; (**c**) waveform of measured strain; (**d**) relationship between measured stress and FEA stress.

To verify the simulated $C_{s}$ and the cyclic stress excited by a unit vibration displacement, as well as to calibrate the stress amplitude control of the ultrasonic fatigue machine, a laser vibrometer (KathMatic KV-HB4525S, Mumuxili Technology company, Nanjing, China) and strain gauges (BE120-1AA, CHENGTEC, Shanghai, China) were employed to measure the vibration displacement and the cyclic stress, respectively. It is worth noting that stress measurement via strain gages should only be taken in a small amplitude and a very short period due to the sensitive grid in the strain gage as well as degradation in fatigue loading. A non-contact measurement that was introduced in our previous work that allowed the quick measurement of a small amplitude via strain gages was sufficient for the stress calibration [29]. Therefore, five measurements with lower amplitudes were tested for calibration. In order to eliminate the heating effect and to enhance the signals of the bending strains, the half-bridge posting method was used in this paper. The strain gages’ attachment on the gauge section of the specimen is shown in Figure 4a. The description of control system and measurement is shown in Figure S3 of Supplementary Materials.

In addition, the excitation displacement at the constraint section was obtained by a laser vibrometer (KV-HB4525S) with an acquisition frequency of 312.5 kHz. Examples of the output waveform on displacement at the excitation section and the strain at the gauge section are presented in Figure 4b,c, respectively. The stress amplitudes from five measurements at different given excitation displacements were plotted as shown in Figure 4d. It was clear that the stress on the specimen had a definite relationship with the excitation displacement. The slope of the fitting was in agreement with that relationship from the simulation. Furthermore, the coefficient of determination revealed that this relationship was linear with the tested system. Therefore, the maximum stress amplitude on the bending specimen could be controlled by the excitation displacement of the ultrasonic fatigue system according to Equation (1). Various stress levels could be obtained by switching the excitation displacement (*A*_0_) of the system; i.e.:

(1) $$\sigma_{a}=C_{s}*A_{0}$$

In addition, it is worth noting that the optimal size of this specimen of VHCF is currently heavily dependent on the material and geometric parameters. The geometric parameters used in this study were designed and shaped on the basis of the TC4 titanium alloy; although such a geometry may not be applicable to all materials, it provided an important geometric structure reference for fatigue testing of the remaining materials.

## 3. Experiment Materials and Methods

### 3.1. Materials

**Figure 5:**
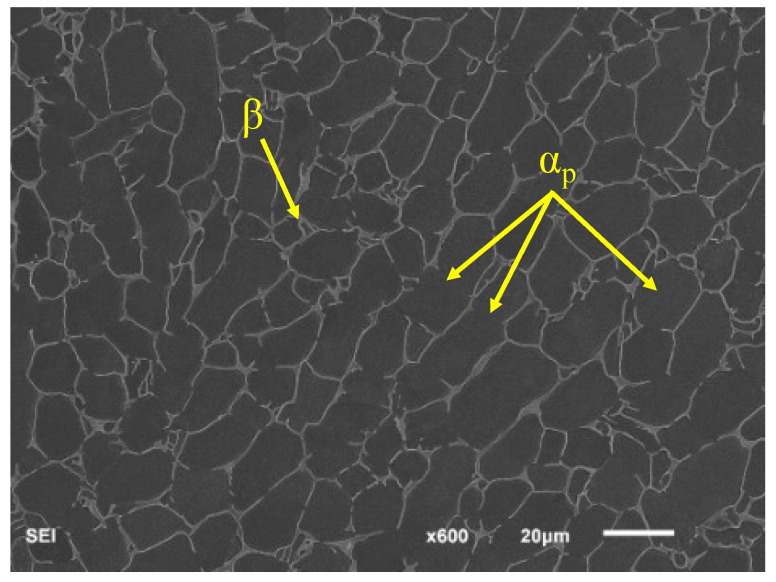
Microstructure of TC4 titanium alloy.

The raw material used in this investigation was an α-β titanium alloy (Ti-6Al-4V) that was supplied as unidirectional rolled bars that were 200 mm in diameter with equiaxed microstructures. The obtained microstructure is shown in Figure 5. Before performing the fatigue experiments, the mechanical properties of the material were tested. The material parameters of the TC4 titanium alloy are shown in Table 1.

### 3.2. Ultrasonic Fatigue Test

**Figure 6:**
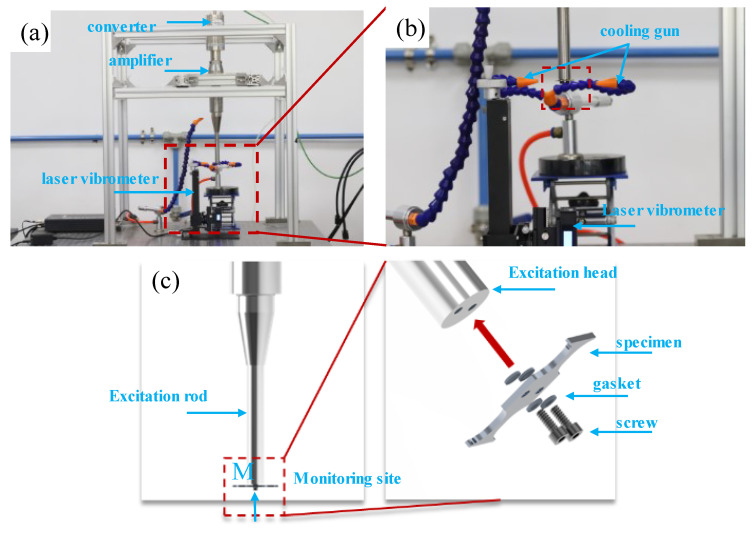
Bending test based on ultrasonic fatigue system: (**a**) full view of the system; (**b**) details of the constraint; (**c**) composition of the bending constraint method.

With the development of ultrasonic technology, ultrasonic vibration loading technology is able to reach very high cycles in a shorter time with the advantage of a 20 kHz or higher frequency. Therefore, an ultrasonic fatigue system with a loading frequency of 20 kHz and a stress ratio of R = −1 was used for this experiment. The ultrasonic fatigue system (TH-UFTS-Pro, Sichuan University, Chengdu, China) mainly consisted of a piezoelectric ceramic transducer, an amplifier, an extension rod, and a laser displacement test system as shown in Figure 6a. The principle of the ultrasonic fatigue loading system was to generate mechanical vibrations through the piezoelectric ceramic transducer, which was used to excite the specimen. When the system was in a resonant state, a displacement field and a stress field were generated along the vibration direction. One of the most important things was to calculate the coefficient ($C_{s}$) when the specimen was loaded. Figures S1 and S2 in the Supplementary Document illustrates the detailed loading principle. Referring to the single thread restraint method for bar specimens, two nuts were used to restrain and fix the thin-plate specimens. The detailed specimen-mounting method is shown in Figure 6c. Figure 6b shows that a laser displacement sensor was added under the excitation section of the specimen that monitored the real-time displacement signal during the entire fatigue experiment and that the displacement amplitude was the excitation displacement (A_0_) of the ultrasonic fatigue system. Figure 6c shows that the test point of the sensor was located at point M.

The small bending fatigue specimens (small mass) did not affect the resonant frequency of the entire ultrasonic fatigue system, and the ultrasonic fatigue test system could run properly [27]. A resonant frequency that was reduced by 40 Hz was considered to be the test-stopping condition; at this time, the fatigue damage of the specimen accumulated enough and the specimen cracked. In addition, when the number of loading cycles exceeded 10^9^, the fatigue test was stopped and the specimen was considered to be undamaged.

### 3.3. Comparison Fatigue Test

**Figure 7:**
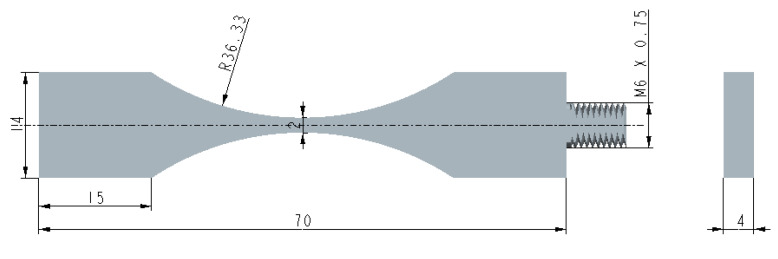
The geometry of the ultrasonic axial loading specimen (unit: mm).

It is well known that the differences in fatigue loading methods lead to differences in experimental results; previous researchers have also conducted corresponding research on different types of loading methods [14,35]. Limited by the impact of loading frequency, the existing research results still remain in the fatigue data for less than 10^7^ cycles. In order to investigate the difference between axial loading fatigue and bending loading fatigue in the VHCF regime, a set of ultrasonic axial loading tests on a plate were conducted for comparison. Figure 7 shows the geometry of the axially loaded specimens.

## 4. Results and Discussion

### 4.1. S-N Curve

**Figure 8:**
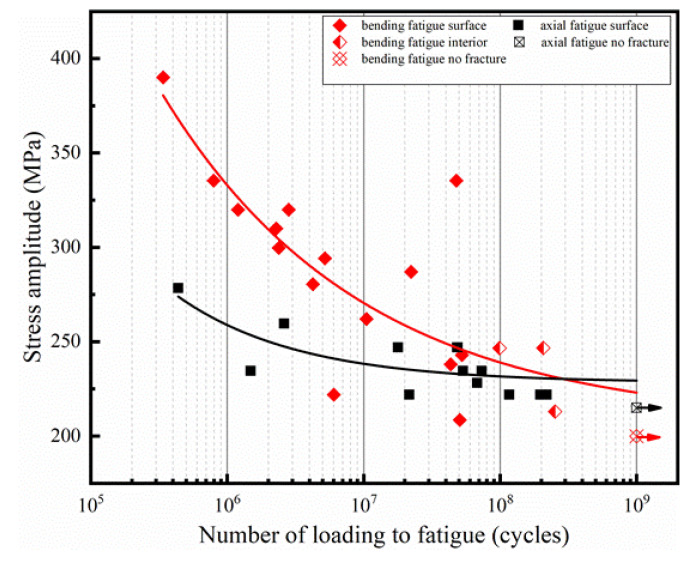
S-N data of the different loading styles involving the bending and axial specimens.

Figure 8 shows the S-N data for the TC4 titanium alloy with different ultrasonic loading types. The solid symbol represents the fatigue crack initiation from the surface, and the semi-solid symbol represents the internal fatigue crack initiation. Red and blue represent the fatigue test results for bending and axial loading respectively. Axially loaded fatigue tests were selected in the range of 200 MPa to 260 MPa, and the stress level was much lower than that in traditional fatigue studies; this result was mainly related to the microstructure [36,37]. Under the axial-loading type, an asymptote S-N curve with a fatigue limit of 235 MPa was observed; when the applied stress level was less than this fatigue limit, the specimens did not suffer fatigue damage even if their cyclic numbers reached 10^9^ cycles, which was consistent with the traditional view of “infinite life” [38]. In addition, under bending loading, there were two typical types of crack initiation; namely, surface crack initiation and internal crack initiation. This was consistent with the results of conventional axial fatigue loading. Finally, the ultrasonic bending fatigue test and the ultrasonic axial fatigue test obtained 19 and 14 valid data, respectively, as shown in Table 2.

The fatigue data of the S-N curve was non-linearly fitted by the classical three-parameter equation [39]:

(2) $${\left(\sigma_{a}-\sigma_{0}\right)}^{a}\times N_{f}=b$$

where $\sigma_{a}$ represents the stress amplitude applied to the fatigue specimen; $N_{f}$ denotes the number of cycles to fatigue failure; and $\sigma_{0}$, a, and *b* are considered as the material constants, which can be solved by the least squares method. The values of $\sigma_{0}$, a, and b of the ultrasonic fatigue specimen were 206.79, 3.38, and 1.26 × 1013, respectively; and the values of $\sigma_{0}$, a, and *b* of the ultrasonic axial fatigue specimen were 235, 7.57, and 3.07 × 1020, respectively.

It can be seen from the fitting curve in Figure 8 that the S-N curve under bending load shows a continuous decline [40,41], and does not show the “step” characteristics of high-strength steel [26,42]. This is consistent with the existing research results. In addition, the S-N curves from bending and axial fatigue crossed near 10^8^ cycles. Previous studies have shown that under high stress amplitude, the bending load can show better fatigue resistance than the axial load. However, the difference will gradually decrease with the increase of cycle times, and even show the trend of intersection [16,21,43].

### 4.2. Fracture Morphology

**Figure 9:**
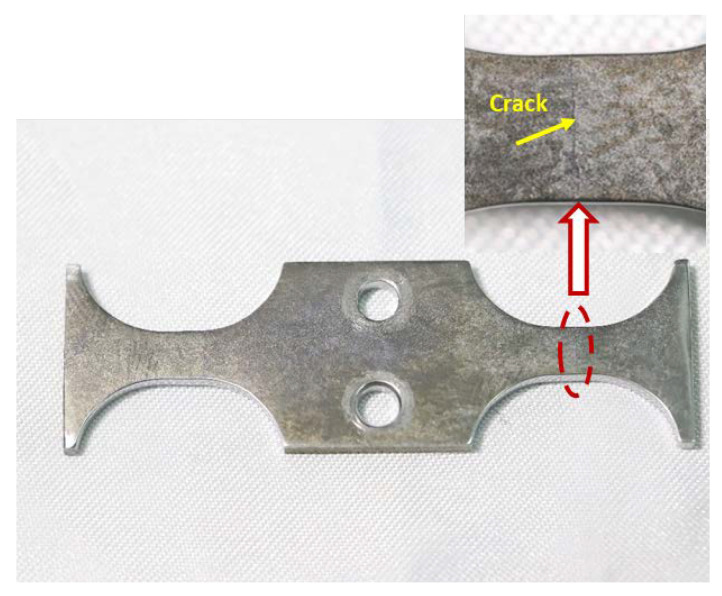
Typical surface crack morphology of specimen failure.

The typical surface cracks of the ultrasonic fatigue specimen when it broke down are shown in Figure 9. It was found that the propagation direction of the continuous cracks on the specimen surface was approximately perpendicular to the stress direction. Meanwhile, the cracks on the specimen surface were located approximately at the center of the gauge section. This experimental phenomenon was consistent with the stress-distribution results along section A-A shown in Figure 3 (the maximum stress of the specimen was located in the center of the gauge section).

**Figure 10:**
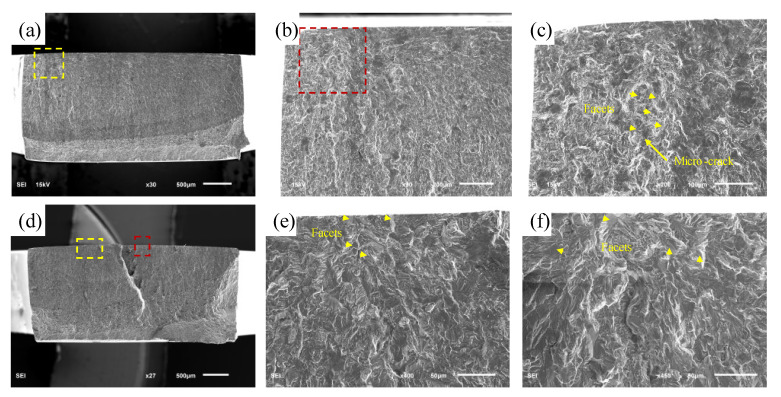
Typical fracture morphology in the HCF: (**a**–**c**) $\sigma_{a}=390.14$ MPa, $N_{f}=3.39\times 10^{5}$ cycles; (**d**–**f**) $\sigma_{a}=220.01$ MPa, $N_{f}=6.05\times 10^{6}$ cycles.

**Figure 11:**
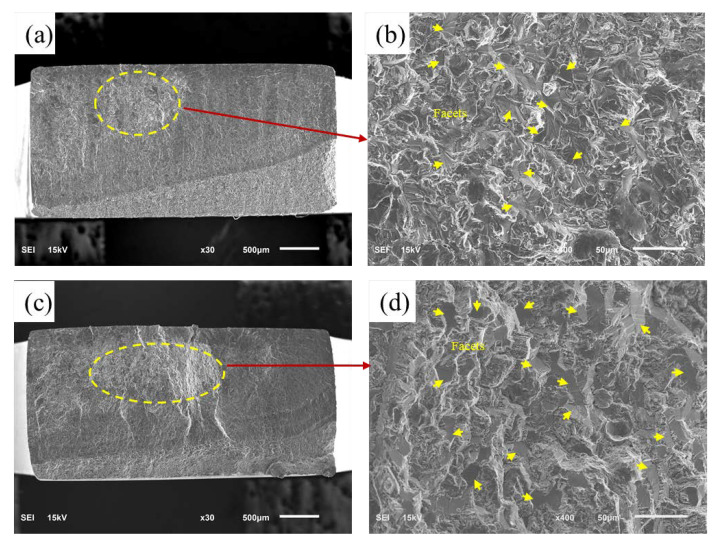
Typical fracture morphology in the VHCF: (**a**,**b**) $\sigma_{a}=246.66$ MPa, $N_{f}=9.91\times 10^{7}$; (**c**,**d**) $\sigma_{a}=213.1$ MPa, $N_{f}=2.53\times 10^{8}$ cycles.

In this study, the fracture surfaces of all specimens were investigated using scanning electron microscopy (SEM), and it was found that many small facets were present in the crack-initiation areas of all fractured specimens. Moreover, two typical fatigue failure modes are summarized and presented in Figure 10 and Figure 11. The typical three-dimensional morphology characteristics of initiation sites are shown in Figures S4 and S5 of Supplementary Materials.

Firstly, Figure 10 shows the typical bending fracture morphology in the HCF regime, including single-point crack initiation and multi-point crack initiation. Figure 10b,e are magnitude views of the area within the yellow dashed box in Figure 10a,d, respectively. Figure 10c,f are magnitude views of the area within the red boxes in Figure 10b,d, respectively. It can be clearly seen that the crack-initiation site is located on the surface of the specimen, and there are some small facets randomly distributed in the initiation area. Several small facets marked with arrows can be clearly seen in the high-magnification morphology of the crack initiation area. The formation of the facets was due to the cleavage of the primary α grains. This failure mode was commonly seen in this study. Similar cases have been reported by other researchers.

Secondly, Figure 11a,c illustrate the typical morphology of internal crack initiation in VHCF. They do not exhibit the fish-eye features typical of axial loading [44,45,46]. High-magnification images of the crack initiation site are shown in Figure 11b,d; there are numerous small facets are indicated by yellow arrows. Other flexural studies have also reported this phenomenon of internal crack initiation [23,33,41]. It is thought that at lower stress amplitudes, the influence of the stress gradient on the bending test is small. Internal flaws in materials currently have a large influence on test results, thereby leading to the phenomenon of internal fatigue crack initiation [20,21].

## 5. Conclusions

Thin-walled components such as blades in turbine engines inevitably suffer the problem of bending vibrations in a large number of cyclic loadings. A very high cycle fatigue study using a similar loading condition will benefit the accuracy of structural integrity design. Rather than the existing ultrasonic fatigue test in axial loading, the current study attempted to provide an alternating loading model for VHCF testing. The main conclusions were drawn as follows:

1. A new bending fatigue model in the resonant state under ultrasonic loading was developed in which the accelerated bending frequency reached 20 kHz and the symmetrical stress distribution at the gauge section was optimized compared with the conventional cantilever model.
2. The S-N curve for the studied material showed that the fatigue data of the bending loading and axial loading intended to have an intersection point when the cycles went beyond 10^8^. Re-discussion of the conservative design strategy may be required regarding bending loading and axial loading in the VHCF regime.
3. There were two types of bending fatigue loading—surface crack initiation and internal crack initiation. As the cycle life increased, the possibility of an internal crack occurring also increased.

It is worth mentioning that the novel ultrasonic fatigue test in this study was strict on the thin-wall sample because the thick plate would also raise the temperature due to friction at the loading section with this accelerated frequency. Therefore, only homogeneous materials were involved in this study; other metals such as additive manufactured and heterogeneous materials require more investigation regarding their feasibility. In addition, the intersection point characteristics in the above S-N curve may not apply to other metals before further study is made on the sensitivity of the microstructure.

## Figures and Tables

**Table 1:** Material parameters of TC4 titanium alloy.

Young’s Modulus (GPa)	Yield Strength (MPa)	Tensile Strength (MPa)	Poisson’s Ratio	Hardness (Hv)
113	915	954	0.33	330

**Table 2:** S-N original data of bending and axial loading specimen.

Bending Load			Axial Load		
Specimen No.	Stress Level (MPa)	Fatigue Cycle (Cycles)	Specimen No.	Stress Level (MPa)	Fatigue Cycle (Cycles)
1	390.14	3.39 × 10^5^	1	278.29	4.38 × 10^5^
2	335.41	4.78 × 10^7^	2	259.49	2.61× 10^6^
3	335.41	7.96 × 10^5^	3	246.96	1.79 × 10^7^
4	320.00	1.20 × 10^6^	4	246.96	4.90 × 10^7^
5	320.00	2.82 × 10^6^	5	246.96	4.84 × 10^7^
6	310.00	2.29 × 10^6^	6	234.43	1.49 × 10^6^
7	299.75	2.39 × 10^6^	7	234.43	5.35 × 10^7^
8	294.25	5.20 × 10^6^	8	234.43	7.36 × 10^7^
9	287.07	2.23 × 10^7^	9	228.16	6.80 × 10^7^
10	280.50	4.25 × 10^6^	10	221.90	1.17 × 10^8^
11	262.10	1.05 × 10^7^	11	221.90	2.16 × 10^7^
12	246.77	2.07 × 10^8^	12	221.90	2.20 × 10^8^
13	246.66	9.91 × 10^7^	13	221.90	1.98 × 10^8^
14	243.02	5.26 × 10^7^	14	215.00	1.00 × 10^9^
15	238.03	4.35 × 10^7^			
16	222.01	6.05 × 10^6^			
17	213.10	2.53 × 10^8^			
18	208.59	5.06 × 10^7^			
19	200.00	1.00 × 10^9^			

## Data Availability

Not applicable.

## Supplementary Materials

The following supporting information can be downloaded at: https://www.mdpi.com/article/10.3390/ma16010005/s1. Figure S1. Sketch of an ultrasonic fatigue system. (a) Vibratory stress and displacement along the system [1] and (b) the flow chart of the control system. Figure S2. bending model: (a) Initial status, (b) Vibration status. Figure S3. (a) strain distribution in the gauge section under 1 μm displacement load constraint; (b) displacement distribution in the gauge section under 1μm displacement load constraint. Figure S4. $\sigma_{a}=390.14$ MPa and $N_{f}=3.39\times 10^{5}$ cycles. Figure S5. $\sigma_{a}=246.66$ MPa and $N_{f}=9.91\times 10^{7}$. References [47,48,49] are cited in the Supplementary Materials.

## Nomenclature

R	Stress ratio (minimum stress/maximum stress)
$\sigma_{a}$	Stress amplitude
$\sigma_{0},\;a,\;b$	Material constants
$N_{f}$	Number of cycles to failure
$C_{s}$	Coefficient of displacement to stress
A_0_	Excitation displacement of the system
R^2^	Coefficient of determination
HCF	High cycle fatigue ($N_{f}$ > 10^5^)
VHCF	Very high cycle fatigue ($N_{f}$ > 10^7^)
FEA	Finite element analysis
