# Supplementary material for: A Novel Ultrasonic Fatigue Test and Application in Bending Fatigue of TC4 Titanium Alloy
Source: Materials (Basel). 2022 Dec 20;16(1):5. doi: 10.3390/ma16010005 (PMC9821665; doi:10.3390/ma16010005)
Supplement: Supplementary file 1 [file materials-16-00005-s001.zip › materials-2065182-supplementary.pdf]

Supplemental Information for materials-2065182

1、The principle of ultrasonic bending fatigue design

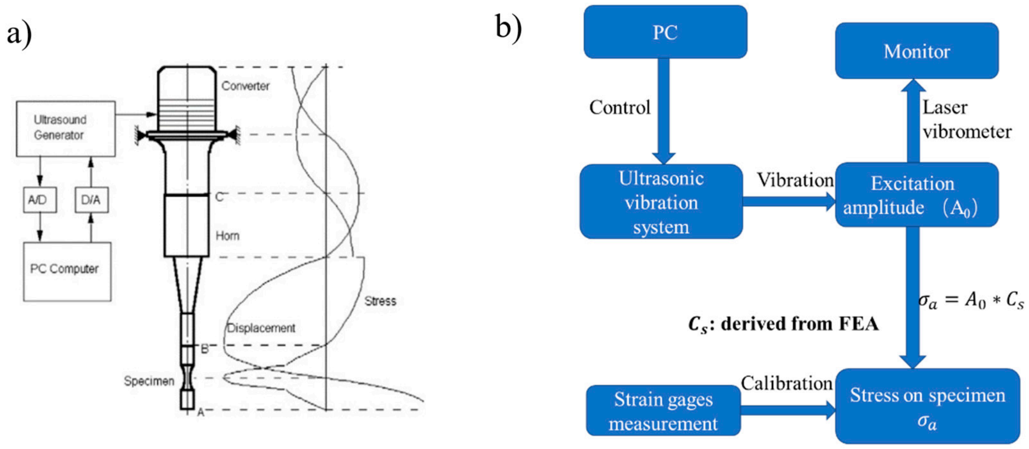

Figure S1. Sketch of an ultrasonic fatigue system. (a)Vibratory stress and displacement along the system[1] and (b) the flow chart of the control system

The fatigue system is mainly composed of an ultrasonic signal generator, piezoelectric ceramic transducer, displacement amplifier, sample, and control system. The principle of the test system is that the ultrasonic signal generator converts 50Hz or 60Hz AC voltage signals into 20 kHz ultrasonic sinusoidal electrical signals, and then the electrical signals are converted into longitudinal mechanical vibration displacement signals with the same frequency by piezoelectric ceramic transducers. When the whole fatigue system is in resonance, the piezoelectric ceramic transducer can generate a corresponding displacement field and stress field in the vibration direction. Finally, through the action of a displacement amplifier, the required strain amplitude of the sample can be obtained. The displacement field and stress field of the piezoelectric ceramic transducer are respectively represented by two curves in Figure S1.

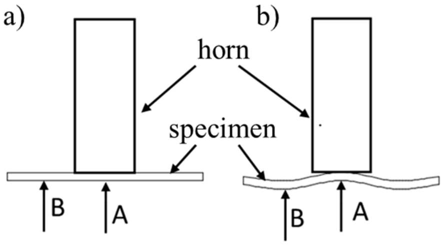

Figure S2. bending model: a) Initial status, b) Vibration status

Theoretically, it is proved that the maximum stress amplitude (σ_{max}) on the middle section of the dog-bone specimen in Figure 1 is in direct proportion to the output displacement amplitude A_0

at the section B (the output end of the displacement amplifier), as shown in equation 1, where C_s is the coefficient stress required for the ultrasonic fatigue test, and the value can be obtained by finite element analysis. Considering the averaging output of insensitive grid to stress distribution along the specimen, even the C_s from the stress calibration is very close to that from FEA, the C_s in the FEA was used during the test.

$$\sigma_{max} = C_s A_0 \tag{S1}$$

As shown in Figure S2, based on ultrasonic axial loading fatigue, the shape and structure of the specimen are redesigned, and the connecting modules required for bending fatigue loading are added, and then a simple very-high cycle bending fatigue test system is constructed. The bending fatigue test system can convert the longitudinal vibration signal into the transverse vibration signal. Under the vibration form of cantilever bending, the maximum stress of the specimen is located at the constraint section (also the excitation section), and the constraint quality at this position will affect the initiation and propagation of bending fatigue crack. Therefore, it is necessary to consider the mode of second-order bending, and transfer the dangerous section in bending loading to the inside of the sample.

2、The strain and displacement diagrams along the A-A path.

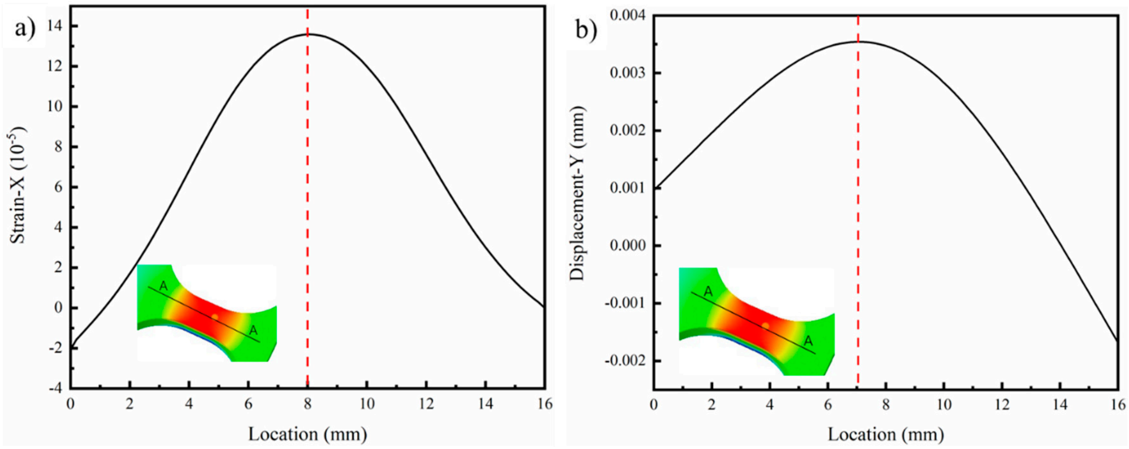

Figure S3. a) strain distribution in the gauge section under 1μm displacement load constraint; b) displacement distribution in the gauge section under 1μm displacement load constraint

3、The morphological characteristics of the fracture surface

In the region of crack initiation, crack extension in the form of short cracks depends on the

sensitivity of the microstructure [2, 3]. It is noteworthy that the length of short cracks is usually the size of one to a few grains in the rough area, while the length of microcracks is usually no more than one grain size. During the accumulation of fatigue loading, when the crack crosses the fisheye region, the crack changes from short crack extension to long crack extension [4]. To illustrate the behavior of short crack extension during crack initiation, the surface profile of the crack initiation region is obtained using laser confocal microscopy along line AB (A is the measurement position and B is the reference position) through the crack initiation region to reveal the crack driving force, as shown in Figure S4 and S5, where the red arrows represent the direction of short crack extension. The fatal short crack extension direction presents different directions relative to the fatigue loading direction, and in Figure S4, the fatal short crack is 42.5° from the loading direction. In Figure S5, the fatal short crack is at 44° with respect to the loading direction. It can be seen that the expansion direction of the short crack is close to 45° with respect to the loading direction, which means that the expansion behavior of the short crack is driven by the maximum shear stress.

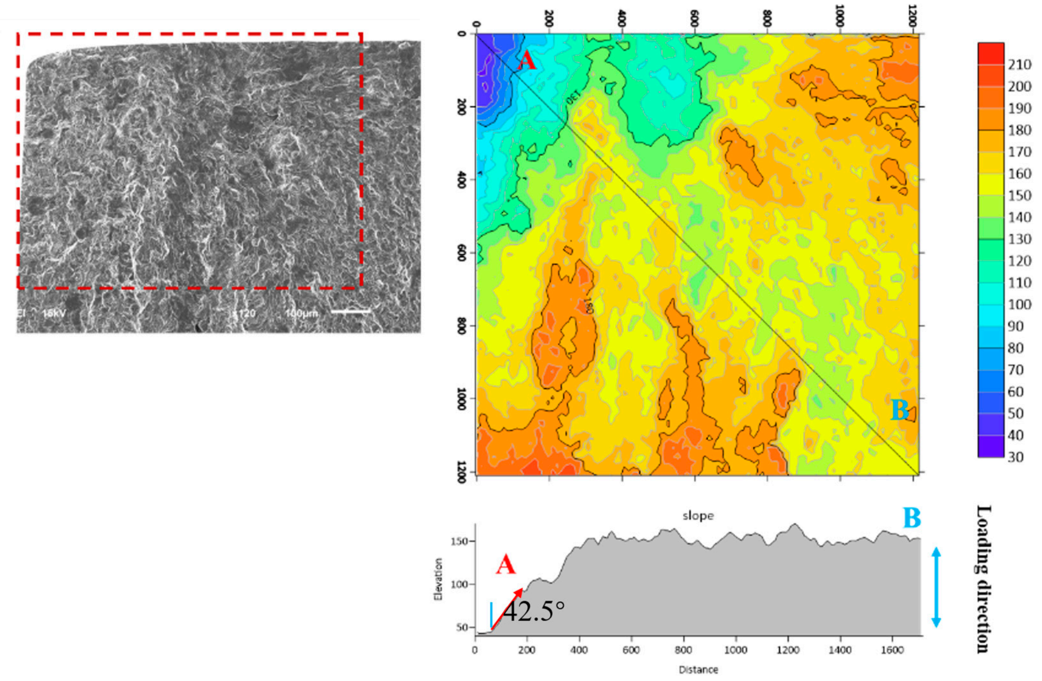

Figure S4. $\sigma_a = 390.14$ MPa and $N_f = 3.39 \times 10^5$ cycles

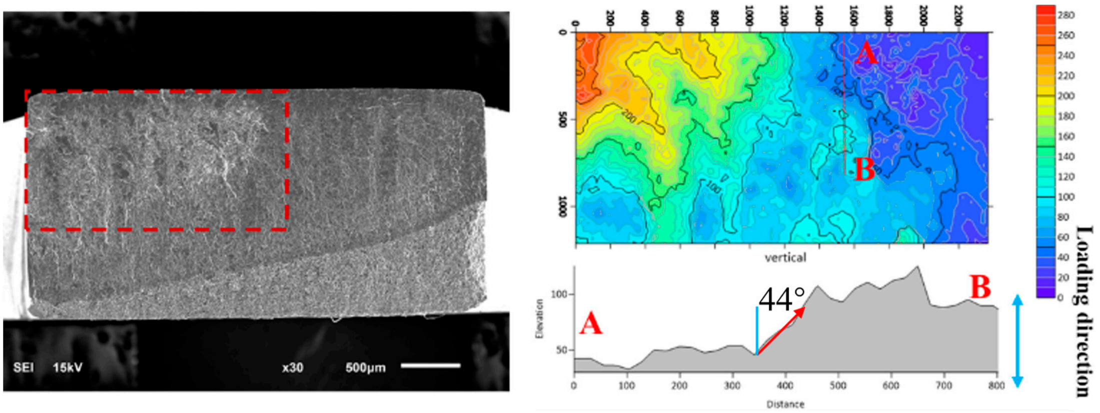

Figure S5. $\sigma_a = 246.66$ MPa and $N_f = 9.91 \times 10^7$

Reference

[1] C. Bathias, Piezoelectric fatigue testing machines and devices, Int J Fatigue 28(11) (2006) 1438-1445.

[2] F. Bridier, D.L. McDowell, P. Villechaise, J. Mendez, Crystal plasticity modeling of slip activity in Ti–6Al–4V under high cycle fatigue loading, International Journal of Plasticity 25(6) (2009) 1066-1082.

[3] D.L. McDowell, F.P.E. Dunne, Microstructure-sensitive computational modeling of fatigue crack formation, Int J Fatigue 32(9) (2010) 1521-1542.

[4] F. Liu, C. He, Y. Chen, H. Zhang, Q. Wang, Y. Liu, Effects of defects on tensile and fatigue behaviors of selective laser melted titanium alloy in very high cycle regime, Int J Fatigue 140 (2020).
